# Highly Stretchable and Transparent Ionic Conductor with Novel Hydrophobicity and Extreme-Temperature Tolerance

**DOI:** 10.34133/2020/2505619

**Published:** 2020-03-19

**Authors:** Lei Shi, Kun Jia, Yiyang Gao, Hua Yang, Yaming Ma, Shiyao Lu, Guoxin Gao, Huaitian Bu, Tongqing Lu, Shujiang Ding

**Affiliations:** ^1^Department of Applied Chemistry, School of Science, Xi'an Key Laboratory of Sustainable Energy Materials Chemistry, MOE Key Laboratory for Nonequilibrium Synthesis and Modulation of Condensed Matter, State Key Laboratory of Electrical Insulation and Power Equipment, Xi'an Jiaotong University, Xi'an 710049, China; ^2^State Key Laboratory for Strength and Vibration of Mechanical Structure, School of Aerospace Engineering, Xi'an Jiaotong University, Xi'an 710049, China; ^3^SINTEF Industry, Forskningsvei 1, 0373 Oslo, Norway

## Abstract

Highly stretchable and transparent ionic conducting materials have enabled new concepts of electronic devices denoted as iontronics, with a distinguishable working mechanism and performances from the conventional electronics. However, the existing ionic conducting materials can hardly bear the humidity and temperature change of our daily life, which has greatly hindered the development and real-world application of iontronics. Herein, we design an ion gel possessing unique traits of hydrophobicity, humidity insensitivity, wide working temperature range (exceeding 100°C, and the range covered our daily life temperature), high conductivity (10^−3^~10^−5^ S/cm), extensive stretchability, and high transparency, which is among the best-performing ionic conductors ever developed for flexible iontronics. Several ion gel-based iontronics have been demonstrated, including large-deformation sensors, electroluminescent devices, and ionic cables, which can serve for a long time under harsh conditions. The designed material opens new potential for the real-world application progress of iontronics.

## 1. Introduction

Distinguished from electronics, iontronics utilize ions contained in electrolytes to implement functions, covering biological ionic systems, electrochemical cells, electrolyte-gated transistors, and electrolyte-based flexible devices [1–5]. The gel electrolyte is a typical solid-state ionic conductor, composed of three-dimensional polymer networks with a large amount of saline solutions or ionic liquids swollen inside the networks [6–8]. Commonly, they are stretchable and fully transparent under visible light. Novel functions have been realized by utilizing gel electrolytes, including electroactive actuators [9–11], stretchable electroluminescent devices [12–14], soft power source [15–17], ionic sensors [18–22], ionic cable [23], and stretchable touch panels [24, 25], which are extremely difficult or even impossible to realize with conventional electronics. For example, Kim's group has demonstrated an ionic touch panel with ultrahigh transparency (98%) and stretchability by using a hydrogel electrolyte [24]. Pan's group has employed ion gels to fabricate a flexible transparent film for interfacial capacitive pressure sensing and supercapacitive nanofabric sensing, leading to ultrahigh mechanical-to-capacitive sensitivity of nF kPa^−1^, which is several orders of magnitude greater than that of the traditional devices [26–28].

However, it is challenging to fabricate gel electrolytes that match the requirements of real-world applications. Our daily life environment has extended humidity (10%~100% relative humidity) and temperature (-40°C~50°C) ranges. To ensure stable operation in engineering applications, devices made from gel electrolytes should bear even harsher conditions. Unfortunately, currently developed stretchable and transparent gel electrolytes cannot satisfy the requirements, which hinder the development of iontronics and their widespread applications. Existing gel electrolytes show unsatisfied humidity stability and quite poor extreme-temperature tolerability. For example, hydrogel electrolytes suffer from water evaporation in open air environment: the large amount of water contained in a hydrogel is easily evaporated at low air humidity and high temperature. Accompanying the loss of water, the transparency, stretchability, and conductivity of the hydrogel electrolyte deteriorate sharply. Moreover, the operating temperature of the hydrogel electrolyte is limited by the freezing and boiling points of water, which is ascribed to their unsustainable characteristics in either a cold or hot environment. Bai's group has enhanced the water retention capacity of hydrogels at low humidity by introducing a highly hydratable salt [29]. Though the resulted hydrogels can absorb a large amount of moisture at high humidity because of the hygroscopicity of the concentrated salts, the hydrogels still cannot prevent water evaporation at high temperature. Recently, Morelle's group has introduced a class of hydrogels that can be cooled to temperature as low as -57°C without being frozen by soaking the gels in CaCl_2_ aqueous solutions [30]. However, at high temperature and high humidity, the prepared hydrogels only present poor stability. Elastomer coating is another strategy to improve the stability of hydrogels under harsh environment with high humidity and temperature [31, 32]. However, it suffers from a complicated manufacturing process, with limited effects on improving stability of the hydrogel electrolyte.

Ion gels have been developed to overcome the shortages of hydrogels [33–36]. They have inherited properties of ionic liquids (ILs), exhibiting unique advantages of neglectable vapor pressure, wide operating temperature range, and broad electrochemical window. However, most existing ion gels are also sensitive to humidity as the ILs easily absorb moisture from air, especially in a high-humidity atmosphere, which will result in swelling and performance degradation of the ion gels, while the existing hydrophobic or air stable ion gels hardly possess good mechanical properties, optical transparency, or extreme-temperature stability [37–39].

Newly developed ionic conducting elastomers possess very low ionic conductivity [40], and organogel ionic conductors have been proven to be unstable in watery environments [41].

Herein, we introduce a hydrophobic and humidity-insensitive ion gel by employing a water-insoluble IL and a hydrophobic polymer network. The ion gel achieves unique combinations of hydrophobicity, humidity insensitivity, high conductivity, high stretchability, excellent transparency, and extreme-temperature tolerance, which can be considered as an ideal material for engineering iontronic devices.

## 2. Results

### 2.1. Design and Synthesis of the Hydrophobic Ion Gels

**Figure 1:**
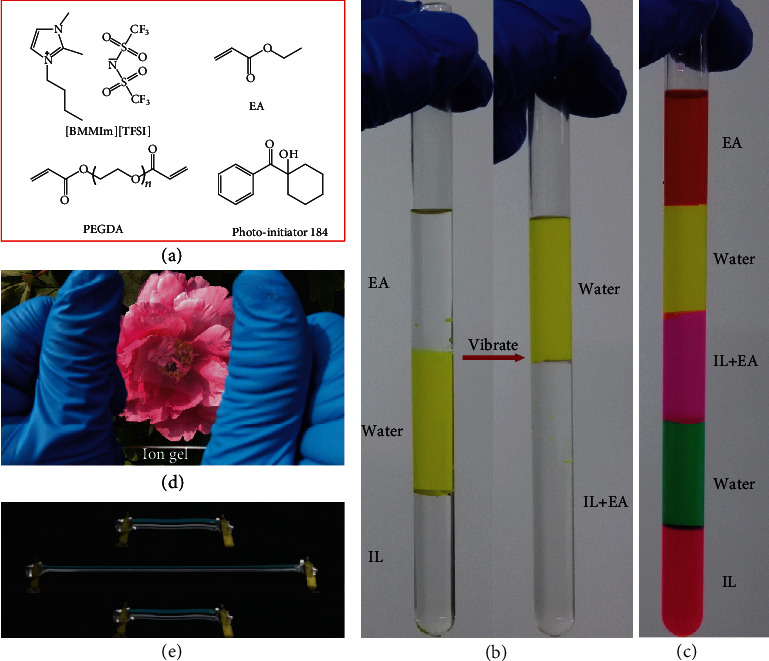
Schematic design of the hydrophobic ion gel. (a) Molecular structure of ion gel precursors: ionic liquid (IL, [BMMIm][TFSI]), polymer monomer EA, and crosslinker PEGDA. (b) Photographs of the designed test-tube experiments, demonstrating the hydrophobicity of EA and IL as well as the compatibility of EA and IL. (c) A colored and layered cocktail made from IL, EA, and water. (d, e) Photograph of a stretched hydrophobic ion gel, demonstrating the excellent transparency and stretchability.

Bearing the above criteria in mind, IL 1-butyl-2,3-dimethylimidazolium bis(trifluoromethylsulfonyl)amine [BMMIm][TFSI] was selected to be the electrolyte salt. It is hydrophobic, colorless, chemically stable, and extreme-temperature tolerant. In other words, it neither dissolves in water nor absorbs large amounts of moisture from the air, and it is fully transparent in the visible light range. In addition, IL [BMMIm][TFSI] possesses an extremely low melting point (-76°C) and an extremely high decomposition temperature (430°C), which makes it sustainable in a wide temperature range. To form a hydrophobic and transparent polymer substrate, ethyl acrylate (EA) was chosen as the monomer to polymerize into poly(ethyl acrylate) (PEA). EA is miscible with IL [BMMIm][TFSI], and its polymerization product, poly(ethyl acrylate) (PEA), is compatible with [BMMIm][TFSI] too; no polymer precipitation or IL separation was observed in the as-prepared ion gel, which ensures the remarkable optical transparency and morphological stability of the ion gel. Additionally, PEA is a typical soft polymer substrate showing good stretchability and rebound resilience, which in turn provides the ion gel good stretchability and rebound resilience. A one-step photo polymerization process was utilized to fabricate the ion gels. The ion gels with desired shape can be prepared in minutes by this process, which facilitates their subsequent applications in various areas, for example, making ion circuits by photolithography. The molecular structure of all the ingredients for preparing the ion gel is shown in Figure 1(a).

The hydrophobicity of IL and EA and their miscibility were tested. Equal amounts of IL, tartrazine aqueous solution, and EA were poured into a test tube, successively. As shown in Figure 1(b), clear interfaces formed between the three layers, indicating that neither IL nor EA is soluble in water. After vibrating the test tube and storing it for a while, IL and EA mixed together and formed a transparent solution, with a clear interface to the water layer. Taking advantage of the miscibility of the components, we designed colorful cocktails. The photograph in Figure 1(c) depicts a layered cocktail made by dyed IL, EA, and aqueous solutions. The clear interfaces between different layers explicitly demonstrated the immiscibility of IL and EA as well as the IL/EA mixture in water, indicating the hydrophobicity of components in the designed ion gel.

The ion gels were prepared by the facile photo polymerization method. First, appropriate amounts of EA (monomer), [BMMIm][TFSI], polyethylene glycol diacrylate (PEGDA) (crosslinker), and 1-hydroxycyclohexyl phenyl ketone (photoinitiator 184) were mixed to form a gelation precursor solution. The molar percentage of the crosslinker and photoinitiator to EA was fixed at 0.1% and 1% throughout the entire study, respectively. For fabricating ion gels with different polymer contents, the volume percentage of EA was set at 20%, 40%, 60%, and 80%. Then, the gelation precursor solution was injected into a transparent glass mold with dimensions of 100 × 100 × 1 mm^3^. Ion gels were cured for 10 minutes by ultraviolet light irradiation (365 nm, 400 W power). Figure 1(d) is a photograph of an as-prepared ion gel under stretching; the ion gel demonstrates excellent optical transparency and mechanical stretchability.

### 2.2. Basic Properties of the Ion Gels

**Figure 2:**
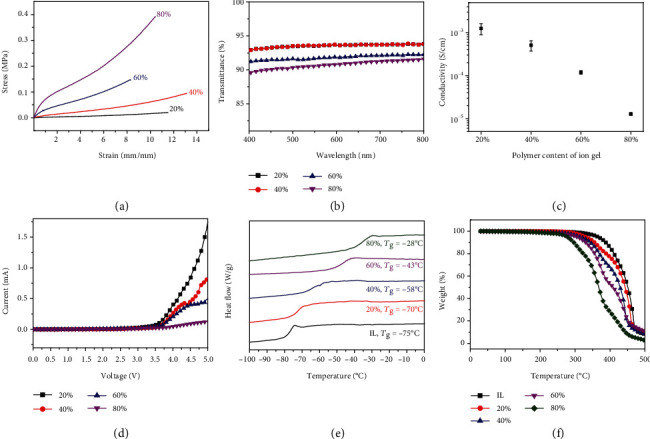
Properties of the ion gels with different polymer contents. 20%, 40%, 60%, and 80% represent the polymer content of the testing samples. (a) Stress-strain curves of the ion gels tested until failure. The elongation at breaks was significantly enhanced compared with existing ion gels. (b) Transmittance versus wavelength curves of ion gels in the visible range; samples for testing were 1 mm thick. (c) Ionic conductivity of the ion gels with different polymer contents. (d) Linear Sweep Voltammetry (LSV) curves of the ion gels with a scanning rate of 1 mV/s. All of the ion gels with different polymer contents showed a high decomposition voltage which exceeded 3.5 V. (e) Differential scanning calorimetry (DSC) curves of the ion gels. The DSC endothermic curve is up. The curves showed they had very low glass transition temperature (*T*_g_), demonstrating low-temperature tolerance. (f) Thermogravimetric curves of the ion gels, demonstrating extremely high thermal stability with decomposition temperature exceeding 300°C.

As shown in Figure 2(a), all the as-prepared ion gels with different polymer contents had extremely high stretchability, with the elongation at break exceeding 800%. The highest stretchability is achieved by the sample of 40% polymer content with the elongation at break of 1312%. Meanwhile, by tuning the polymer content of the ion gels, a sample with distinct mechanical properties can be obtained. With increasing polymer content, the ion gel obtained a higher Young's modulus and an enhanced breaking strength. The mechanical properties of the ion gel can also be adjusted by tuning the crosslinker content. Increasing the crosslinker content leads to a lower elongation at break, a higher Young's modulus, and a higher breaking strength (Figure S1). A cyclic loading-unloading test was also performed. The samples of ion gels were loaded with strain up to 500% with a loading rate of 100 mm/min; the strain was immediately unloaded after it approached 500%. The ion gels fully recovered to their original lengths after unloading of the strain, and the loading and unloading curves almost overlap (Figure S2), indicating fully reversible mechanical properties and negligible hysteresis.

Benefiting from the good compatibility between the polymer network PEA and the solvent [BMMIm][TFSI], the ion gels possess excellent transparency. As shown in Figure 2(b), in the entire visible light range, the transmittance of all the samples (1 mm thick) was above 90%. Samples with 20% and 40% polymer contents had the highest transmittance (93.6% at 550 nm). As the polymer content increases, the transmittance of the ion gels reduced slightly. The excellent transparent property was critically important for optical iontronics, such as touch panels and electroluminescent devices.

The ion gels exhibited good ionic conductivity. Figure 2(c) depicts conductivities of ion gels with 20%, 40%, 60%, and 80% polymer contents. At 20°C, the conductivities of ion gels were 1.25 × 10^−3^, 5.08 × 10^−4^, 1.19 × 10^−4^, and 1.28 × 10^−5^ S/cm, respectively. As a typical ionic conductor, the ion gels showed impedance-frequency dependency (Figure S3a) as well as phase angle-frequency dependency (Figure S3b). Meanwhile, all the ion gels exhibited high decomposition voltage. Linear Sweep Voltammetry curves are depicted in Figure 2(d). It is worth noting that the decomposition voltages of all ion gels exceeded 3.5 V, demonstrating a wide electrochemical window. In other words, the operating voltage applied between the ion gels can reach up to 3.5 V, while for hydrogels, the applied voltage usually cannot exceed 1 V. A higher decomposition voltage resulted in a higher electric stability of ionic conductors.

More importantly, the ion gels showed extreme-temperature tolerance. The glass transition temperatures (*T*_g_) were measured by differential scanning calorimetry (DSC). The designed ion gel is a composite of PEA and [BMMIm][TFSI]; therefore, *T*_g_ is affected by the content of the two components. *T*_g_ of pure PEA was -22°C, which is much higher than that of [BMMIm][TFSI] (-75°C). As canbe seen from Figure 2(e), *T*_g_ of the ion gel decreased successively with decreasing polymer content, owing to the extremely low *T*_g_ of the ionic liquid [BMMIm][TFSI]. The lower the PEA content the lower the *T*_g_. All the ion gels possess extremely low *T*_g_, indicating that they are still elastic even at low temperature, and as long as the operating temperature is higher than *T*_g_, they sustain high elasticity. Moreover, the stability of ion gels at high temperature was investigated by thermogravimetry. Figure 2(f) shows that the ion gels possessed ultrahigh decomposition temperatures, indicating a stable working temperature up to 200°C.

The ion gels also exhibited good hydrophobicity. Figure S4 shows the results of contact angle measurements of ion gel films. Water droplets gradually spread out on the ion gel surfaces without permeating into the ion gel substrates, demonstrating that the ion gels were incompatible with water.

### 2.3. Temperature Characteristics of the Ion Gel

**Figure 3:**
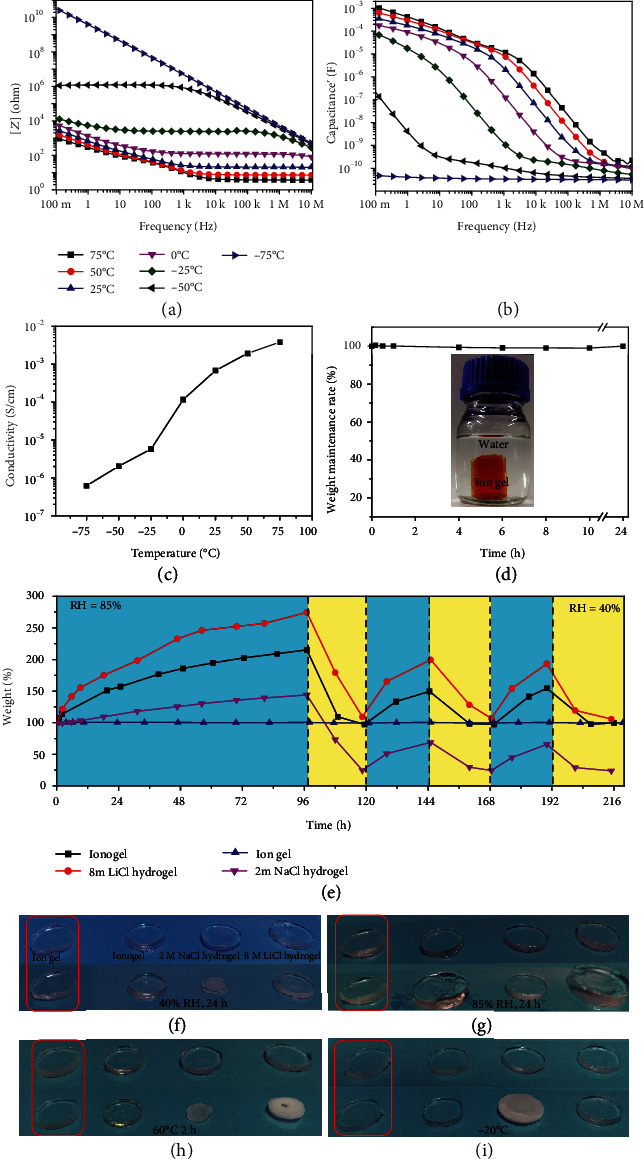
Characteristics of the ion gel (40% polymer content). (a) Impedance magnitude (∣*Z*∣) versus frequency plots over a wide temperature range. (b) Capacitance′ versus frequency plots over a wide temperature range. (c) Conductivities of the ion gel over a wide temperature range. (d) Weight retention rate versus time plot of the ion gel stored in a large amount of water, demonstrating the water resisting property. Its weight showed almost no change all along the testing time, and the ion gel maintained its original shape. (e) Weight retention rate versus time plot of the several kinds of ionic conductors stored at different relative humidities (RH), demonstrating the humidity stability of the ion gel. Testing temperature was 25°C. Blue area represents RH = 85%; yellow area represents RH = 40%. (f) Photograph of the ionic conductors before (upper) and after storing at 40% RH for 24 hours. (g) Photograph of the ionic conductors before (upper) and after storing at 85% RH for 24 hours. (h) Photograph of several kinds of ionic conductors before (upper) and after heating at 60°C for 2 h. (i) Photograph of the ionic conductors before (upper) and after storing at -20°C.

Furthermore, we measured the electrical properties of the ion gel at various testing temperatures. Unless otherwise stated, the sample used had 40% polymer content. A 1 mm thick sample was sandwiched between two copper electrodes (diameter of 30 mm) for impedance spectroscopy tests. Temperature change affected the alternating current impedance properties of the ion gel.  Figure 3(a) shows impedance magnitude (∣*Z*∣) versus frequency curves at different temperatures. At -75°C, a temperature below *T*_g_ of the ion gel (-58°C), the ∣*Z*∣ versus frequency curve was similar to that of a dielectric material VHB 4910 (Figure S5). As the temperature rose above *T*_g_, the curves showed the typical feature of ionic conductors, indicating that the ion gel behaved as an ionic conductor. The higher the temperature the lower the ∣*Z*∣. The capacitance versus frequency plots showed a similar variation trend. As shown in Figure 3(b), when the temperature decreased from 75°C to -75°C, the capacitance sharply decreased in the whole frequency range (0.1 Hz–10 MHz). At -75°C, the ion gel behaved as a dielectric material; the ion gel did not show electric double layer capacitance (EDLC) at low frequency, similar to VHB 4910 (Figure S6). The dielectric constant and dielectric loss versus frequency plots of the ion gel at -75°C are given in Figure S7; the ion gel showed a dielectric constant of about 5.4 in the whole frequency range. When the temperature was higher than -50°C, the gel behaved as an ionic conductor, the capacitance was super huge at low frequency, and it decreased sharply as the frequency increased, because at high frequency, the ions' movement was unable to match the switching of electric field and it became more difficult to form EDLC, resulting in a low capacitance. The phenomenon can be explained as follows: when the temperature was below *T*_g_, polymer chain and IL were frozen, the ions contained in the ion gel can hardly move in response to external electric field, and as a result, ionic conductivity was lost and EDLC cannot form between the gel and the electrode. As shown in Figure 3(c), ionic conductivity of the ion gel increased several orders of magnitude as the temperature increases. At a low temperature of -50°C, the ion gel kept an ionic conductivity of 2.05 × 10^−6^ S/cm, and at 75°C, the value was 3.82 × 10^−3^ S/cm, making it applicable in both very cold and hot environments.

### 2.4. Hydrophobicity and Humidity Stability of the Ion Gel

More importantly, the ion gel possesses unique hydrophobicity and humidity insensitivity. A dyed ion gel (40% polymer content) was kept in water for 24 h, and its weight change was followed carefully. The weight maintenance rate curve is shown in Figure 3(d). The weight of the ion gel had been quite stable in the investigated time span, indicating the high stability of the ion gel in water environment. The dyed ion gel did not swell or shrink in water by visual observation, and it maintained its original shape after being stored in water for 24 h. We compared the humidity sensitivity of several kinds of ionic conductors, including normal hydrogel (2 M NaCl hydrogel) [23], water retention hydrogel (8 M LiCl hydrogel) [28], ionogel [33], and our ion gel. The samples were first hydrated at a high relative humidity of 85% for 96 hours, followed by dehydrating at a low relative humidity of 40% for 24 hours; the hydration and dehydration cycle was repeated twice subsequently with storage time at each RH level of 3 hours. The weight retention rate of the testing samples stored at different relative humidity (RH) levels was recorded, and the results are depicted in Figure 3(e). Obviously, except for the ion gel, the ionic conductors showed remarkable weight change as the RH changed. At a high RH of 85%, 8 M LiCl hydrogel, ionogel, and 2 M NaCl hydrogel absorbed a large quantity of moisture from the air, resulting in weight gain of 275%, 215%, and 143% after 96 h, respectively. And for the ion gel, the value is below 0.5% (Figure S8). The subsequent storage at low RH induced serious weight loss in these three samples. In the following hydration/dehydration processes, the weight change of these three samples was not as great as that in the first cycle, which implies that these three samples were humidity sensitive with RH greatly affecting their weights. On the contrary, the as-prepared ion gel kept stable weight throughout the whole testing process. The weight change of the gels also influences and reflects their morphology. Figures 3(f) and 3(g) show the morphology change of the samples at different RH. Apparently, all but the ion gel's morphology was affected by humidity.

**Figure 4:**
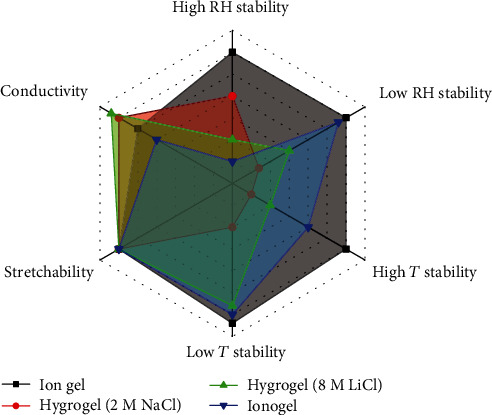
Relative plot of the properties of several ionic conductors.

In order to investigate the extreme-temperature tolerance of the materials, they were stored in an oven and a refrigerator for high- and low-temperature stability measurements, respectively. The most remarkable change was observed in hydrogels. As shown in Figure 3(h), both the 2 M NaCl hydrogel and 8 M LiCl hydrogel dried at 60°C with the 2 M NaCl hydrogel showing obvious shrinkage and turbidity. Though the volume change of the 8 M LiCl hydrogel was not as significant as that of the 2 M NaCl hydrogel, its transmittance deteriorated after the thermal treatment. When they were cooled to -20°C and stabilized for 2 hours, the 2 M NaCl hydrogel was frozen and turned white as shown in Figure 3(i), whereas no obvious change in transmittance and volume was observed in the 8 M LiCl hydrogel, contributing to its colligative property. In contrast to the hydrogels, both our ion gel and ionogel were stable at high and low temperatures, keeping the same appearances (volume and transmittance) after being treated under harsh temperatures.  Figure 4 is the relative plot of the properties of the several ionic conductors; our designed ion gel covered the maximum area, indicating that the material possesses excellent comprehensive performance among the existing ionic conductors.

### 2.5. High-Performance Iontronics Based on As-Prepared Ion Gel

**Figure 5:**
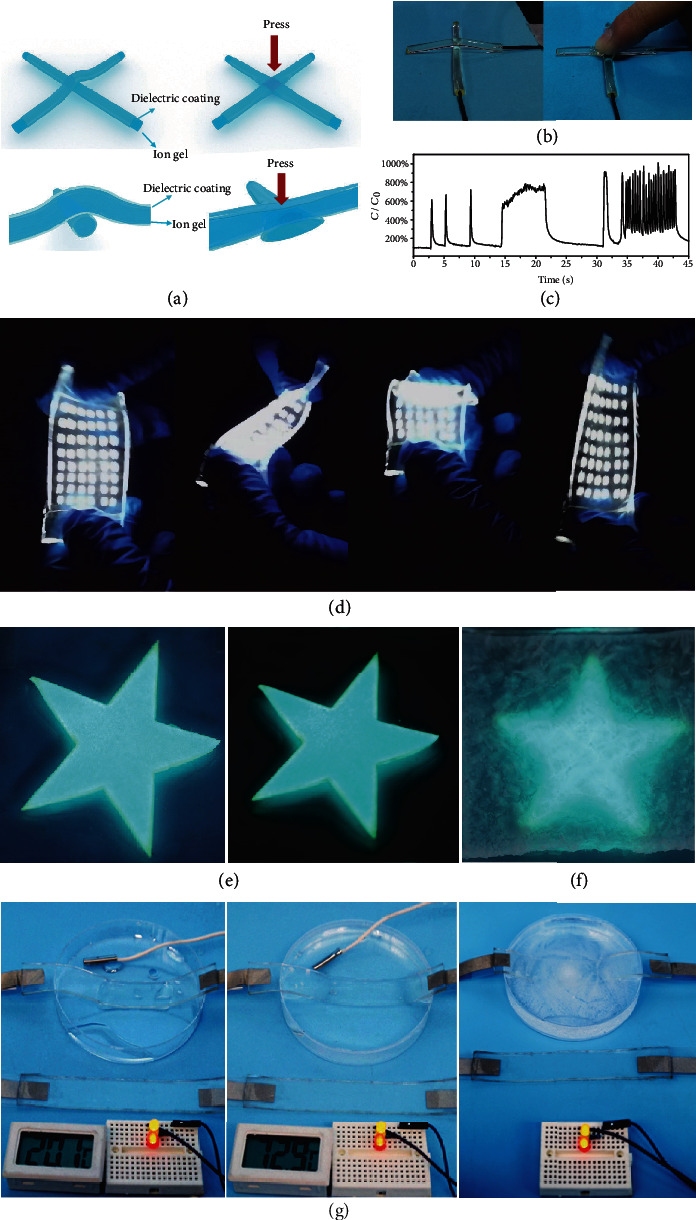
Ionic devices based on ion gel. (a) Scheme of the capacitive press sensor based on ion gel. (b) Photographs of cylindrical ion gel capacitive sensor. The gel electrodes were coated with transparent insulating rubber layer PDMS. (c) Capacitance change versus time plot with different press stimuli. (d) Photographs of ion gel-based flexible LED device. (e) Photographs of ion gel-based electroluminescent device, original (left) and after storing (right) in air for 1 month. (f) Photograph of hydrogel-based electroluminescent device after storing in air for 1 day; the device lost uniformity of luminescence as well as flexibility. (g) Ion gel cable in water environment at different temperatures; the ion gel cable was quite stable at such harsh conditions.

Several ionic devices were developed using the as-prepared ion gel. Figure 1(e) and Figure S9 show photographs and sensing properties of the ion gel. By virtue of the high stretchability of the ion gel, the resistance variation can reach up to several folds, which is impossible to achieve using traditional electronic conductors. Supplementary Movie 1 shows the resistance change under different stretch stimuli. Taking advantage of the deformability of the material, we also developed a capacitive press sensor (Figures 5(a)–5(c) and Supplementary Movie 2). The cylindrical ion gel was coated with a transparent insulating rubber layer (polydimethylsiloxane, PDMS). Two of the coated ion gels were crossed to form a variable capacitor, with the ion gel serving as a deformable electrode and the coatings as the dielectric layer. When the intersection was pressed, the shape of the ion gel electrodes was changed, leading to an increased effective overlap area of the capacitor, which induced the increase of capacitance. When the force was removed, the capacitor recovered to its original shape, accompanied with recovered capacitance. The recovery time is longer than the response time; the phenomenon is because of the lightly sticky property of the coating silicone rubber. Supplementary Movie 3 and Figure 5(d) display a flexible light-emitting diode (LED) based on the ion gel. The ion gel served as a flexible transparent conductive substrate; the device was lightened by alternating current with a frequency of 500 Hz. Supplementary Movie 4 and Figure 5(e) exhibit an electroluminescent device using the ion gel as electrodes, in which the electroluminescent layer (ZnS : Cu in PDMS, 0.1 mm thick) was sandwiched between ion gel layers. When an alternating voltage with a frequency of 1 kHz and a peak value of 3.3 kV was applied to the two ion gel electrodes, the device emitted bright luminescence. After leaving the device in the open air in the lab for 1 month, no observable change was noticed in either its morphology or luminescent property. On the contrary, the hydrogel-based electroluminescent device lost uniformity of luminescence and flexibility after being stored in open air for 1 day (Figure 5(f)). Finally, we demonstrated the application of the ion gel as a cable in harsh conditions. As shown in Figure 5(g), the cable was immersed in water at indicated temperatures. The ionic cable could still transfer electric energy to lighten the LEDs even at temperatures above 70°C or below 0°C.

## 3. Discussion

The designed ion gel possesses unique characterization of hydrophobicity, humidity insensitivity, wide working temperature range, high conductivity, considerable stretchability, and high transparency, which is among the best-performing ionic conductors ever developed for flexible iontronics.

## 4. Materials and Methods

### 4.1. Synthesis of the Ion Gels

Firstly, ionic liquid [BMMIm][TFSI] (99%, Linzhou Keneng Material Technology Co. Ltd., China), monomer EA (99%, Aladdin), crosslinker PEGDA (average Mn 575, Sigma-Aldrich), and photoinitiator 184 (98%, Aladdin) were intensively mixed to form a transparent precursor solution. Then, the solution was injected into a release film-coated glass mold. After being irradiated with ultraviolet light (365 nm, 400 W power) for 10 min, the ion gel was cured. The molar percentage of photoinitiator 184 to EA was 1% throughout the entire experiment, and the crosslinker content was varied from 0.1% to 1% (molar percentage to EA). Different polymer content samples were synthesized by adjusting the volume ratio of [BMMIm][TFSI] and EA. For example, the precursor composition of a typical 40% polymer content ion gel was as follows: 20 ml EA (0.188 mol), 0.384 g photoinitiator 184 (1% molar percentage to EA), 0.216 g PEGDA (0.2% molar percentage to EA), and 30 ml [BMMIm][TFSI]. Obtained ion gels were put in a vacuum drying oven at 100°C for 2 h to remove the stench.

### 4.2. Synthesis of Hydrogels for Comparison

2 M NaCl hydrogel was synthesized by thermally initiated polymerization: 2.84 g acrylamide (monomer), 0.046 g ammonium persulfate (initiator), 0.012 g N,N′-methylenebisacrylamide (crosslinker), and 2.34 g NaCl were dissolved in 20 ml water to form a precursor solution. After injecting the precursor solution into a glass mold, the mold was covered with a plastic film to avoid water evaporation. The mold was then put into an oven and kept at 60°C for 3 h to cure the hydrogel. The synthesis of 8 M LiCl hydrogel was similar to that of 2 M NaCl hydrogel, except for replacing the 2.34 g NaCl with 9.66 g LiCl·H_2_O.

### 4.3. Synthesis of Ionogel for Comparison

Precursor solution was prepared with the following: 1-ethyl-3-methylimidazoliumethylsulfate (IL, 90% volume), acrylic acid (monomer, 10% volume), PEGDA (crosslinker, 0.6 mol % of monomer), and photoinitiator 184 (1 mol % of monomer). Then, the solution was injected into a release film-coated glass mold. The ionogel was cured by ultraviolet light (365 nm, 400 W power) irradiating for 10 min.

### 4.4. Characterization

*Mechanical tests*: dumbbell-shaped samples with testing measure of 12.0 × 2.0 × 2.0 mm^3^ were tested on an electronic tensile machine (CMT6503, MTS) with a 50 N load cell. The stretching rate was set at 100 mm min^−1^.

*Transparency tests*: transmission mode of an UV-Vis spectrophotometer (PE Lambda950, Instrument Analysis Center of Xi'an Jiaotong University) was performed to measure the transmittance with air as reference. The specimens have a thickness of 1 mm.

*Decomposition voltage tests*: samples were sandwiched by two round steel electrodes to measure the decomposition voltage via Linear Sweep Voltammetry (LSV) on an electrochemical workstation (CHI660E) with a scan rate of 0.5 mV s^−1^.

*Impedance tests and ionic conductivity calculation*: the impedance tests at various temperatures were performed on a broadband dielectric/impedance spectrometer (Novocontrol GmbH). Testing Vrms (voltage effective value) was set at 1 V. Conductivity was calculated by the equation *σ* = *L*/*SR*, where *L* is the thickness of the material, *S* is the effective overlap area, and *R* is the bulk resistance (read from the Nyquist plot).

*Differential scanning calorimetry (DSC) measurements*: the DSC measurements were performed by using aluminum crucible on Mettler Toledo Star system (DSC822e) via a scanning rate of 10°C min^−1^ from -100°C to 0°C under flowing N_2_.

*Thermogravimetric analysis (TGA) measurements*: the TGA measurements were performed by using alumina crucible on a TGA Q 5000 via a scanning rate of 10°C min^−1^ from room temperature to 500°C under flowing N_2_.

*Water resistive stability test*: Sudan III dyed cylindrical ion gel (40% polymer content) sample with a diameter of 12 mm and height of 15 mm was put in a bottle with about 80 ml water. The weight of the ion gel was recorded at proper intervals.

*Humidity stability tests*: proper amount of water was injected into a plastic box (26 × 26 × 20 cm) to cover the whole bottom of the box to create a high-humidity environment (85% RH). The box was kept at room temperature for several hours till the inside humidity was stabilized. The gel samples with dimensions of 40 × 20 × 2 mm^3^ accompanied with a humidity sensor were put into the box. The gels were weighed at certain time intervals. Low humidity environment (40% RH) was created by using a moisture ejector in an oven.

### 4.5. Ion Gel-Based Large-Deformation Sensors

By using transparent PTFE pipe as the mold, elongated cylindrical ion gels were prepared. The material with that shape served as the large-deformation resistive sensor; its resistance was detected by an LCR meter (TH2832) at a frequency of 10 kHz (to minimize the influence of EDLC). A large-deformation capacitive sensor was fabricated by using elongated cylindrical ion gels with PDMS (SYLGARD 184 silicone elastomer) coating as electrodes. The coating method was as follows: ion gels were dipped into a PDMS precursor solution with a composition of a base and crosslinker with a ratio of 10 : 1. The ion gels were then hung in an oven at 60°C for 6 h to form the coating.

### 4.6. Electroluminescent Devices

Electroluminescent powder ZnS (Shenzhen Obest) was mixed in the PDMS precursor with a weight ratio of 1 : 1. Then, the precursor was slicked by using a scraper to get a fixed height of 0.1 mm. The electroluminescent layer was cured in an oven at 60°C for 6 h. The electroluminescent layer was sandwiched by two ion gel layers (1 mm thick) afterwards to form the electroluminescent device. For electroluminescent tests, the applied frequency is 1 kHz with a voltage peak value of 3.3 kV.

## Data Availability

The data that support the findings of this study are available from the corresponding author, upon reasonable request.

## Supplementary Materials

Supplementary 1 Figure S1: stress-strain curves of the 20% polymer content ion gels tested until failure. Figure S2: mechanical loading-unloading test of the ion gels with different polymer contents. Figure S3: impedance properties of ion gels with different polymer contents. Figure S4: photographs of contact angle test of the ion gels. Figure S5: impedance properties of VHB 4910 and ion gels at different temperatures. Figure S6: capacitance′ versus frequency plot of VHB 4910 at 25°C. Figure S7: dielectric constant and dielectric loss versus frequency plots of the 40% polymer content ion gel at -75°C. Figure S8: weight retention rate versus time plot of the ion gel storing at a high RH (85%). Figure S9: resistance change versus time plot with different stretch stimuli.

Supplementary 2 Movie 1: ion gel-based resistive sensor under different stretch stimuli.

Supplementary 3 Movie 2: ion gel-based capacitive sensor under different stretch stimuli.

Supplementary 4 Movie 3: a flexible LED based on ion gel.

Supplementary 5 Movie 4: an electroluminescent device using ion gel as electrodes.
