# Supplementary material for: Highly Stretchable and Transparent Ionic Conductor with Novel Hydrophobicity and Extreme-Temperature Tolerance
Source: Research (Wash D C). 2020 Mar 19;2020:2505619. doi: 10.34133/2020/2505619 (PMC7520821; doi:10.34133/2020/2505619)
Supplement: Supplementary 1 — Figure S1: stress-strain curves of the 20% polymer content ion gels tested until failure. Figure S2: mechanical loading-unloading test of the ion gels with different polymer contents. Figure S3: impedance properties of ion gels with different polymer contents. Figure S4: photographs of contact angle test of the ion gels. Figure S5: impedance properties of VHB 4910 and ion gels at different temperatures. Figure S6: capacitance′ versus frequency plot of VHB 4910 at 25°C. Figure S7: dielectric constant and dielectric loss versus frequency plots of the 40% polymer content ion gel at -75°C. Figure S8: weight retention rate versus time plot of the ion gel storing at a high RH (85%). Figure S9: resistance change versus time plot with different stretch stimuli. [file 2505619.f1.docx]

Supplementary Materials

Figure S1. Stress-Strain curves of the 20% polymer content ion gels tested until failure.Figure S2. Mechanical loading-unloading test of the ion gels with different polymer contents. Figure S3. Impedance properties of ion gelss with different polymer contents. Figure S4. Photographs of contact angel test of the ion gels. Figure S5. Impedance properties of VHB 4910 and ion gels at different temperatures. Figure S6. Capacitance’ verses frequency plot of VHB 4910 at 25 ^o^C. Figure S7. Dielectric constant and dielectric loss verses frequency plots of the 40% polymer content ion gel at -75 ^o^C. Figure S8. Weight retention rate versus time plot of the ion gel storing at a high RH (85%). Figure S9. Resistance change versus time plot with different stretch stimuli.


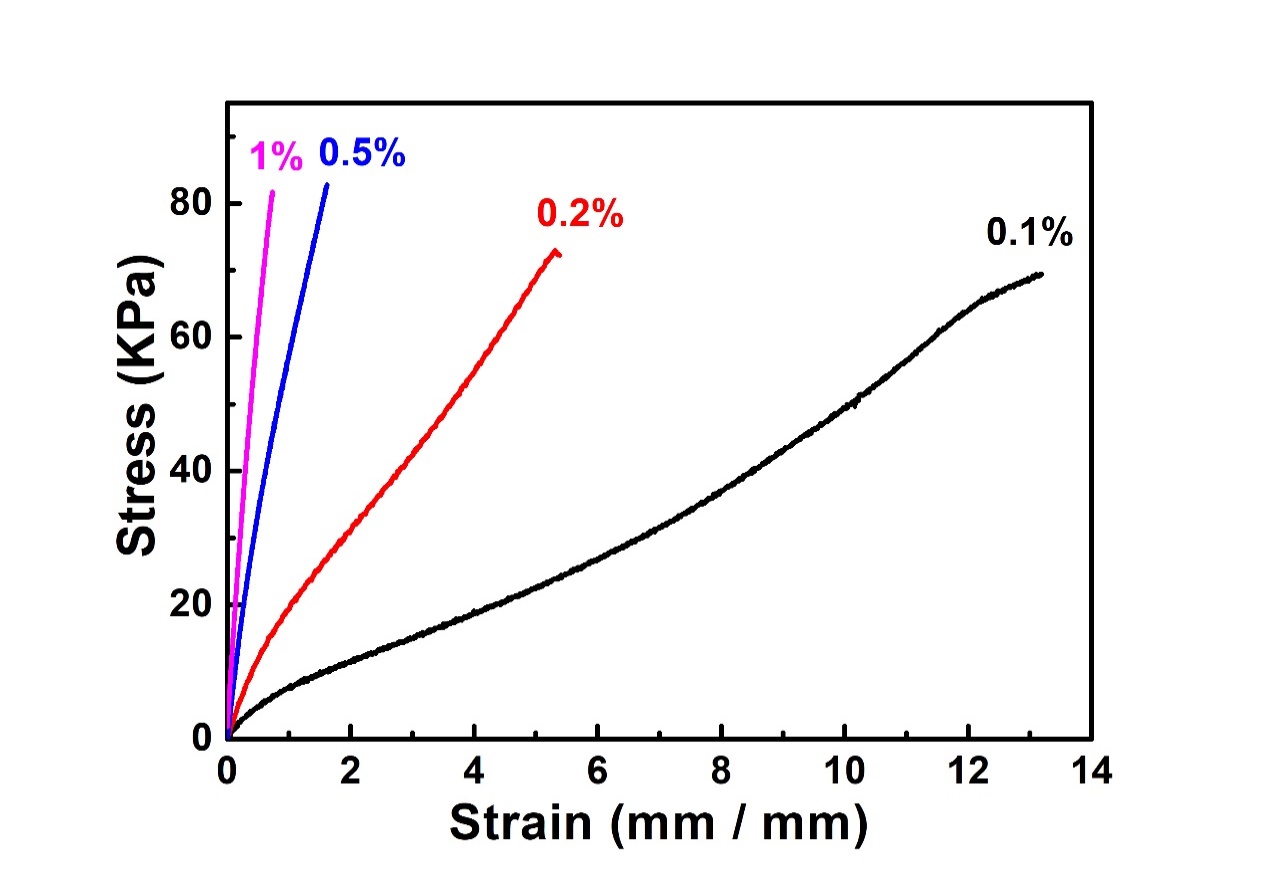


Figure S1. Stress-Strain curves of the 20% polymer content ion gels tested until failure. 0.1%, 0.2%, 0.5% and 1% represent the crosslinker content (molar ratio to monomer).


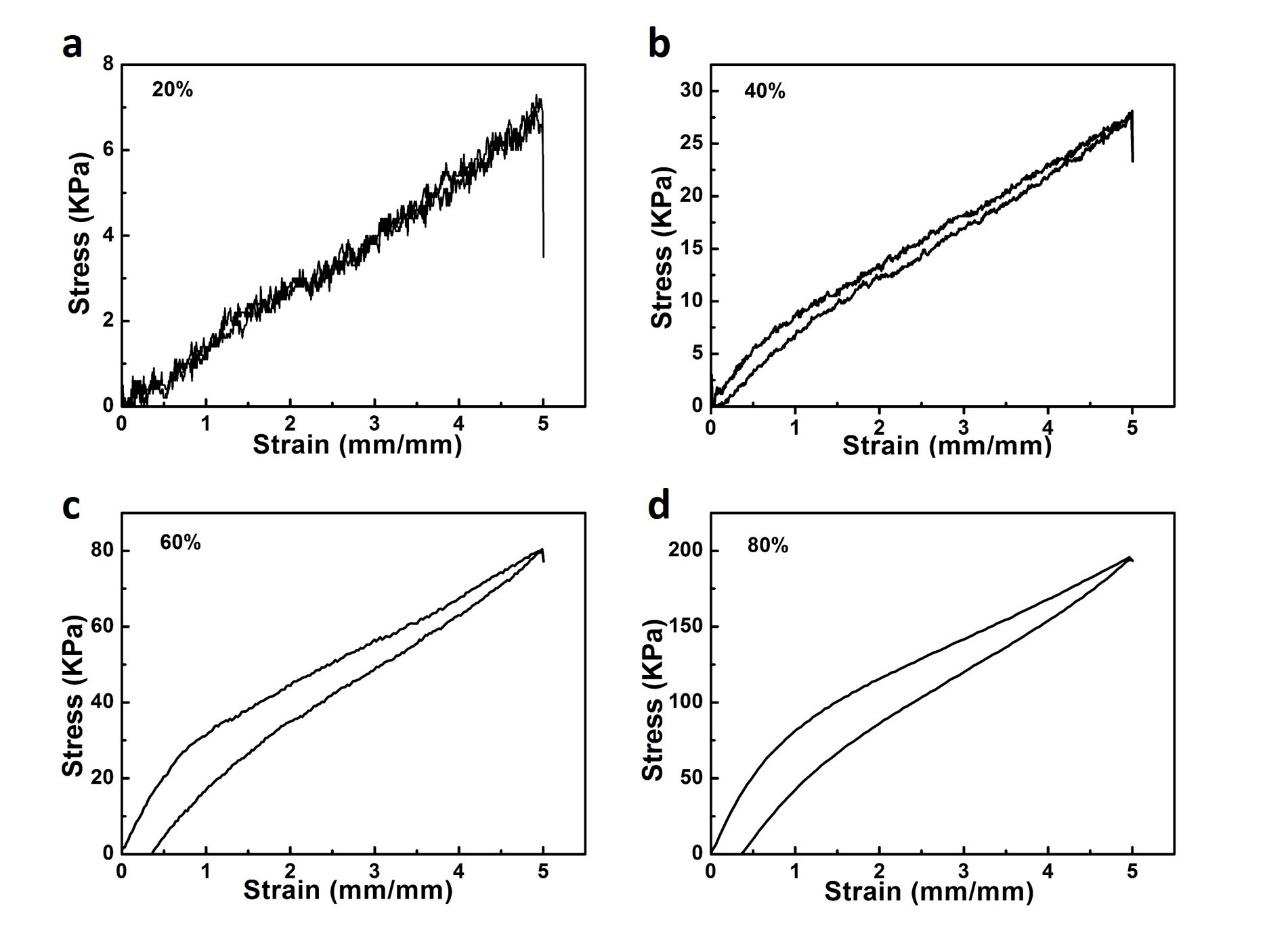


Figure S2. Mechanical loading-unloading test of the ion gels with different polymer contents.


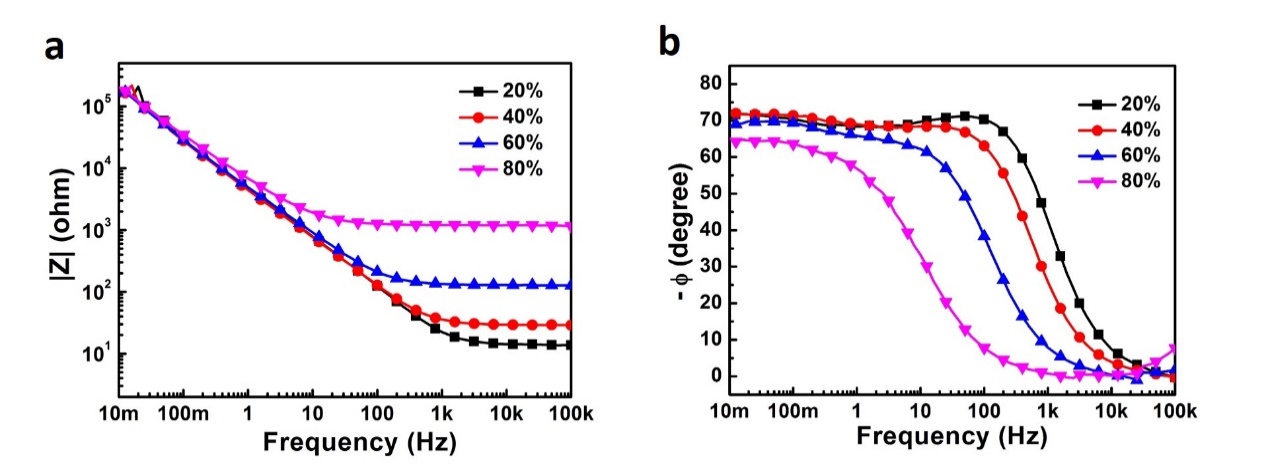


Figure S3. Impedance properties of ion gelss with different polymer contents. a) Plots of impedance magnitude (|Z|) versus testing frequency. b) Plots of phase angle (ϕ) versus testing frequency.


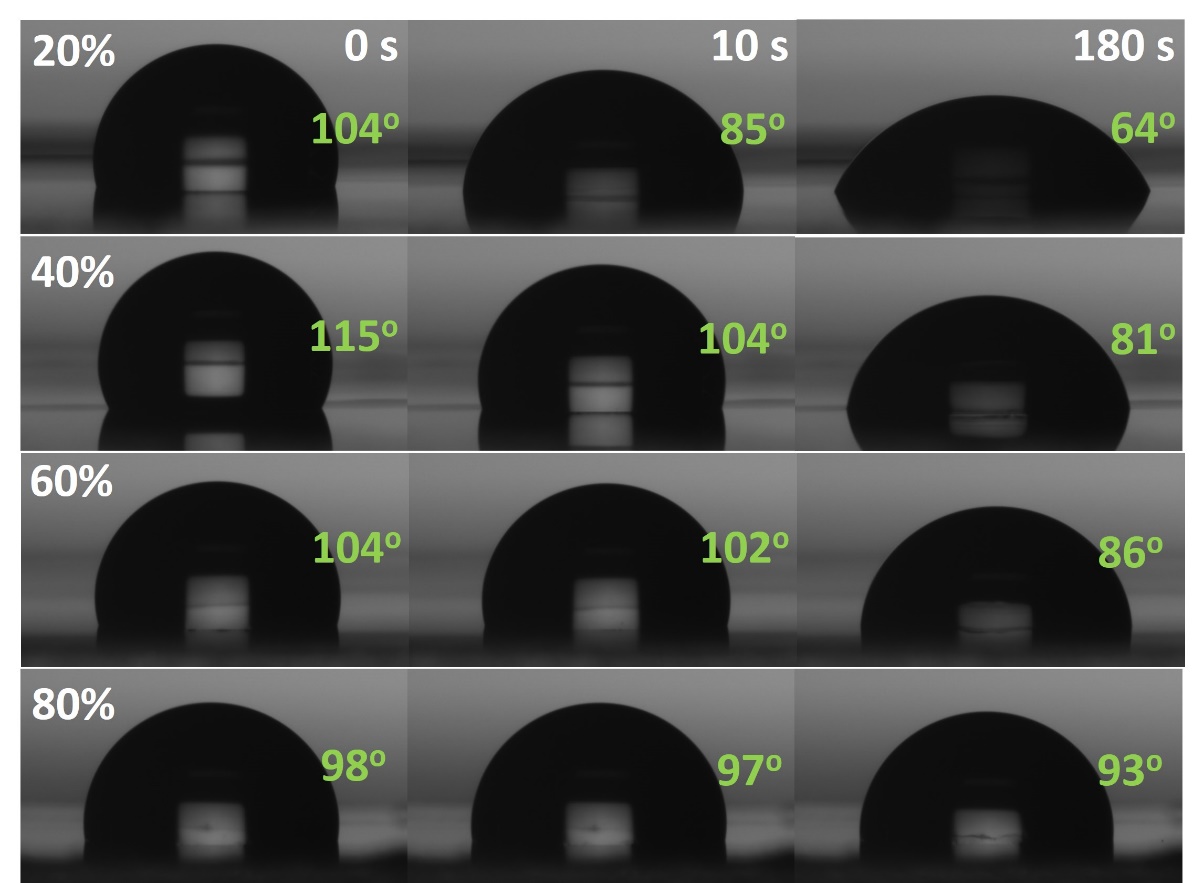


Figure S4. Photographs of contact angel test of the ion gels. 0s, 10s, 180s represent the time after the water droplet falls, the green number represent the contact angel.


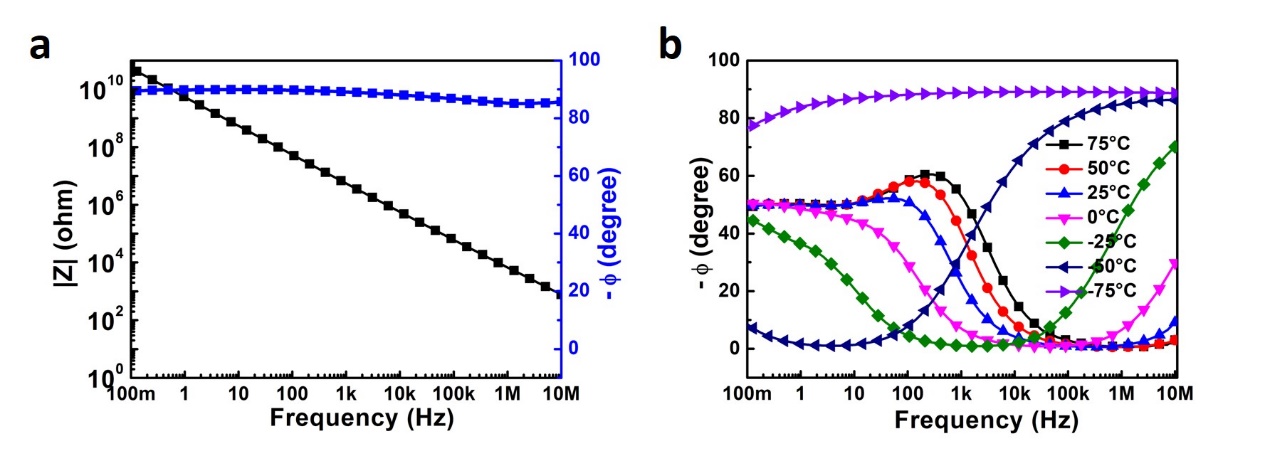


Figure S5. a) Plot of impedance magnitude (|Z|, black line) and negative phase angle (−ϕ, blue line) versus testing frequency of VHB 4910 at 25 ^o^C. b) Negative phase angle (−ϕ) versus frequency plots of the 40% polymer content ion gel over a wide temperature range.


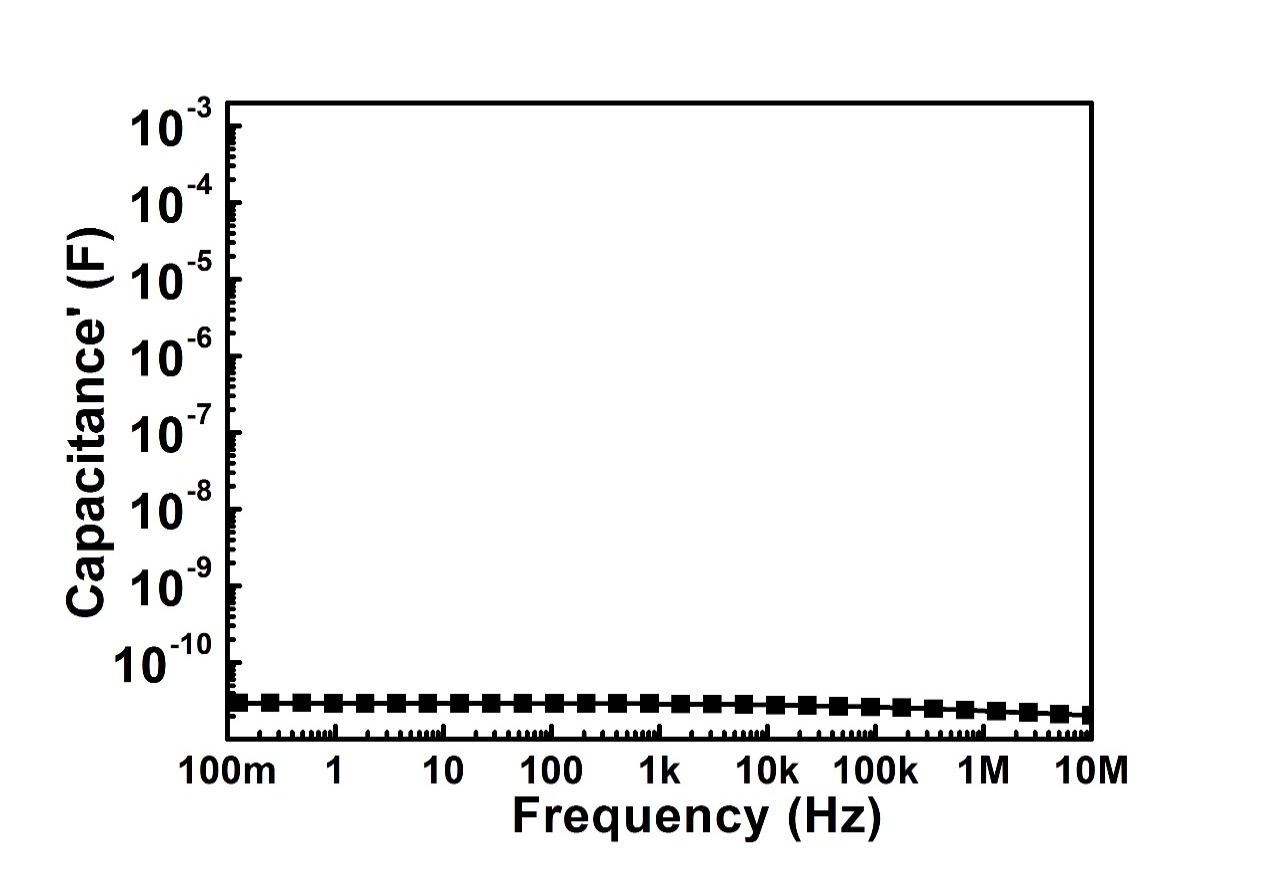


Figure S6. Capacitance’ verses frequency plot of VHB 4910 at 25 ^o^C.


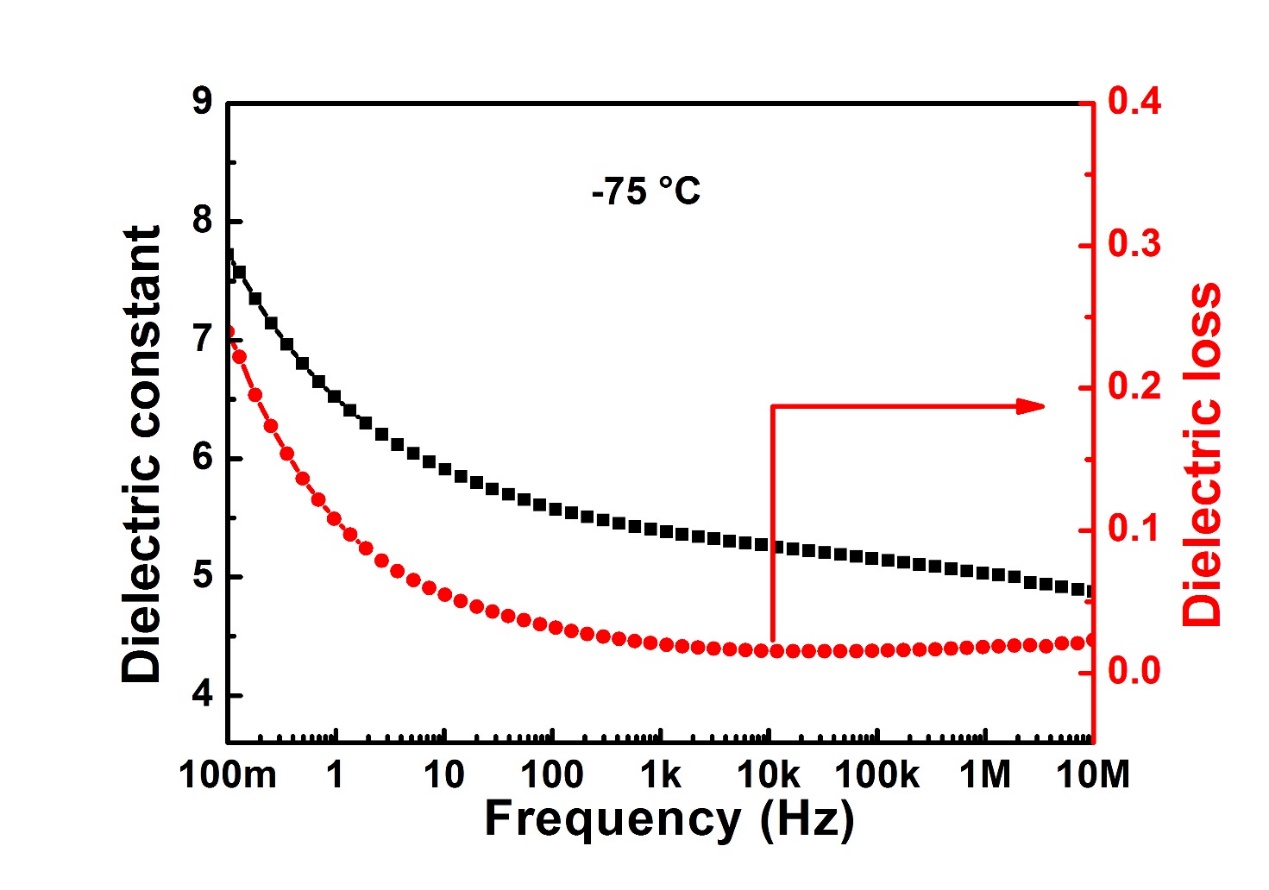


Figure S7. Dielectric constant and dielectric loss verses frequency plots of the 40% polymer content ion gel at -75 ^o^C.


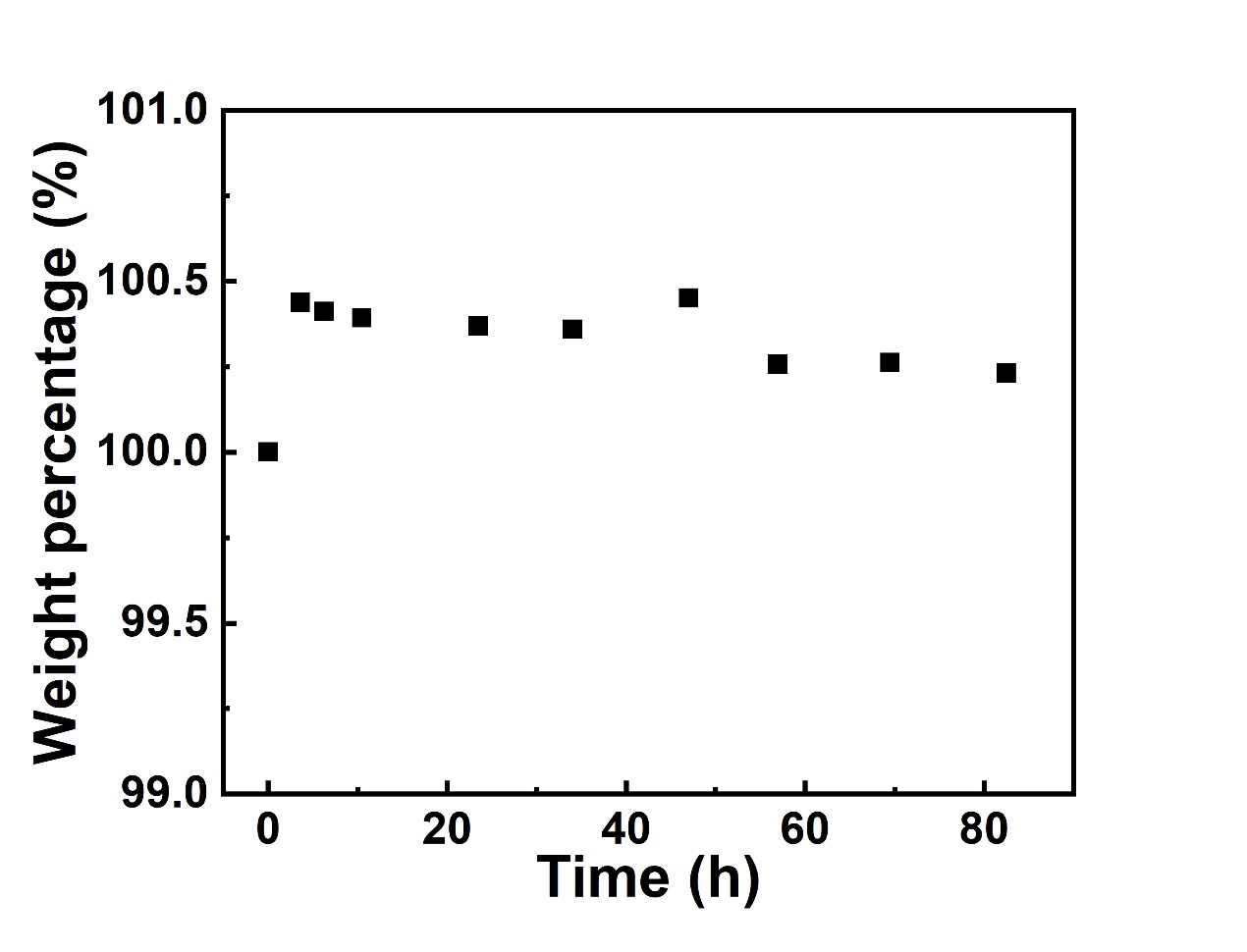


Figure S8. Weight retention rate versus time plot of the ion gel storing at a high RH (85%), demonstrating of the humidity stability of the ion gel. Testing temperature was 25 ^o^C.


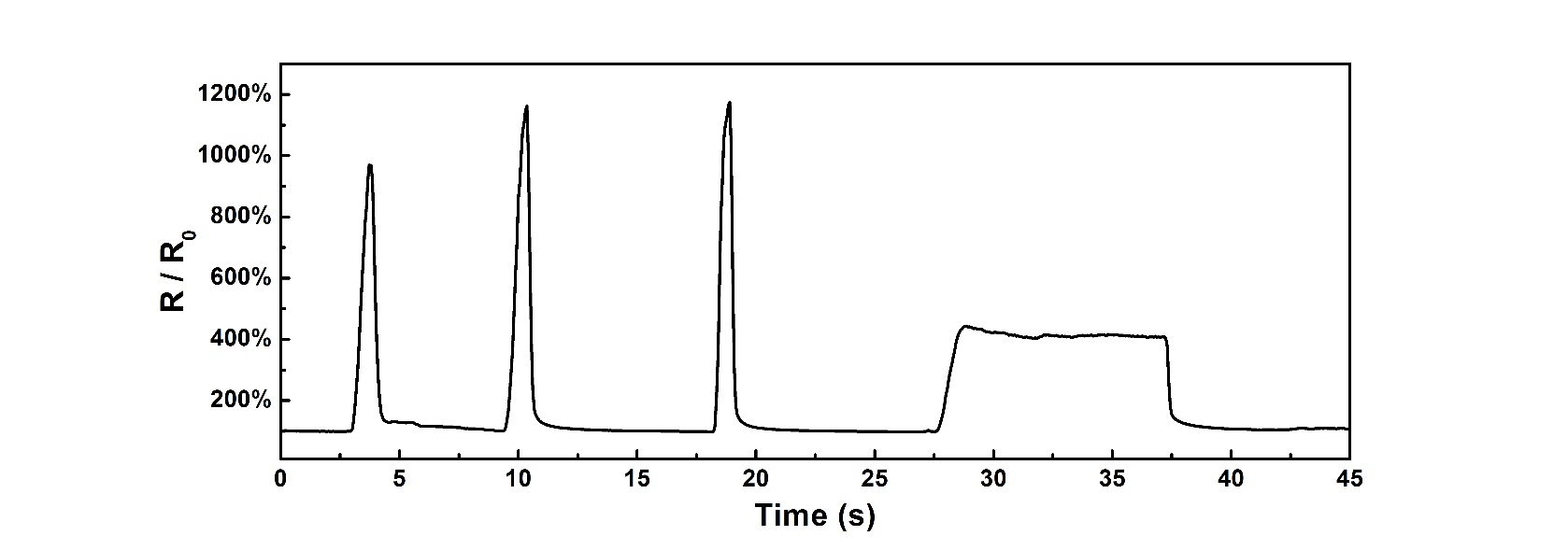


Figure S9. Resistance change versus time plot with different stretch stimuli.
